# Patients with rare endocrine conditions have corresponding views on unmet needs in clinical research

**DOI:** 10.1007/s12020-021-02618-z

**Published:** 2021-02-03

**Authors:** Johan P. de Graaf, Friso de Vries, Anne Dirkson, Olaf Hiort, Alberto M. Pereira, Márta Korbonits, Martine Cools, on behalf of Research and Science Work Package of Endo-ERN

**Affiliations:** 1 Dutch Pituitary Foundation, Nijkerk, The Netherlands; 2 Department of Medicine, Division of Endocrinology and Centre for Endocrine Tumors Leiden (CETL), Leiden University Medical Centre, Leiden, The Netherlands; 3 Leiden Institute for Advanced Computer Science, Leiden University, Leiden, The Netherlands; 4 Division of Paediatric Endocrinology and Diabetes, Department of Paediatrics and Adolescent Medicine, University of Lübeck, Lübeck, Germany; 5 Centre for Endocrinology, William Harvey Research Institute, Barts and The London School of Medicine and Dentistry, Queen Mary University of London, London, EC1M 6BQ UK; 6 Department of Paediatric Endocrinology, Ghent University Hospital, and Ghent University, Ghent, Belgium

**Keywords:** Endo-ERN, Rare disease, Patient perspective, Research, Endocrinology

## Abstract

**Purpose:**

European Reference Network on Rare Endocrine Conditions' (Endo-ERN) mission is to reduce and ultimately abolish inequalities in care for patients with rare endocrine conditions in Europe. This study assesses which themes related to rare endocrine conditions are prioritized by patients for clinical research.

**Methods:**

A survey was developed, translated into 22 different European languages, and distributed to patients with rare endocrine conditions. Patients were asked to give priority scores to listed prespecified topics: fertility, heritability, tiredness, daily medicine intake, sleep quality, physical discomfort, and ability to work, partake in social life, and sports. They were also asked to suggest further important areas for research in open fields.

**Results:**

After data cleaning, 1378 survey responses were analyzed. Most responses were received from Northern (47%) and Western Europeans (39%), while Southern (11%) and Eastern Europe (2%) were underrepresented. Respondents were most interested in research concerning ability to participate in social life and work. Patients suggested key areas to work: long-term side effects of medical treatments and quality of life. Some priorities differed between disease groups, both for prespecified and open topics and reflected aspects of patients’ individual conditions.

**Conclusions:**

With this large survey, Endo-ERN gained insight into patients’ unmet needs in scientific research. Patients prioritized research on ability to work and participation in social activities, though needs differ between the disease groups. Clinical experts should incorporate the results of this survey into the design of future studies on rare endocrine conditions. We aim to utilize these results in designing patient-reported outcome measures for the disease areas covered by Endo-ERN.

## Introduction

In 2017, 24 European Reference Networks (ERNs) were installed with the mission to reduce health care inequalities for all patients with rare and/or complex conditions across the European Union. This is to be achieved through cross-border expert consultation and guideline conformity enabling the highest standard of care [1]. The European Reference Network on Rare Endocrine Conditions (Endo-ERN), at present, is the largest ERN, and comprises 86 reference centres representing 26 countries. These reference centres are specialized in endocrine services, selected by Endo-ERN based on strict criteria, and acknowledged as centres of expertise by the EU. Endo-ERN has defined its mission in five work packages (WPs): Education and Training, Electronic-Health and Information and Communication Technology, Research and Science, Quality of Care and Patient View and Diagnostics and Laboratory analysis, and has identified eight organ- and physiology-based main thematic groups (MTGs): Adrenal, Disorders of calcium and phosphate homoeostasis, Genetic disorders of glucose and insulin homoeostasis, Genetic endocrine tumour syndromes, Growth and genetic obesity syndromes, Pituitary, Sex development and maturation and Thyroid (see the Endo-ERN website for a figure on the structure of Endo-ERN) [2, 3].

The Research and Science WP aims to facilitate research in the field of rare endocrine conditions and provide recommendations to Endo-ERN members about relevant research questions. Another important task of this WP is to identify available research infrastructure and clinical trial opportunities and facilitate their translation into feasible research projects relevant to paediatric and adult endocrine fields. These projects are then prioritized based on scientific quality, relevance and available budget [4].

Involvement of patients is crucial to all ERNs. Endo-ERN integrated patient representation already during the construction phase of its network, with equal responsibilities for patient representatives and health care providers. Each MTG and WP is co-chaired by two endocrinologists, specialized in paediatric and adult endocrinology, respectively, and at least one patient representative. All patient representatives are endorsed by their national organizations and have been approved by Eurordis, the European umbrella organization for rare disease patient organizations, and officially installed as European Patient Advocacy Group representative. These representatives ensure the presence of the patients’ voice within the Network.

In recent times, patient involvement in the management of their disease has become increasingly important. A notable example is shared decision making, where physicians provide all options and existing evidence to patients and together both parties decide on the preferred approach in the specific setting and circumstances. Disease outcomes, however, have mostly been identified and studied by physicians so far. With the rise of value-based health care, more studies have considered patient-reported outcome measures, such as quality of life. However, the opinion of patients on prioritized research topics is generally unknown and patient involvement in decisions on research initiatives is minimal. Therefore, when providing patients with all available evidence, information on topics that are crucial to patients may be lacking. This study aims to identify unmet needs in medical research from a patient perspective, focusing on rare endocrine diseases. In addition, it aims to identify information resources preferred by patients in order to gain access to most recent scientific information related to their disease.

## Methods

### Construction of the survey

A survey containing 14 questions was constructed by the Research and Science WP and Endo-ERN chairs. The survey was thereafter assessed on content and understandability by research specialists of Eurordis and PGO Support (a public funded facilitator for patient organizations in the Netherlands) whose comments and advice were used to adjust the survey. The survey was translated on a voluntary basis by patient advocates identified through the Endo-ERN and Eurordis networks that were native speakers of the target language into 22 different European languages: Bulgarian, Croatian, Czech, Danish, Dutch, English, Estonian, French, German, Greek, Hungarian, Islandic, Italian, Lithuanian, Macedonian, Norwegian, Polish, Portuguese, Slovakian, Slovenian, Spanish and Swedish. The survey translators were invited to provide suggestions on the survey questions. The survey was distributed via heath care providers affiliated to Endo-ERN, patient representatives, national patient advocacy groups and via the Orphanet newsletter. The survey was constructed with EUSurvey, a survey tool provided by the European Commission, available at https://ec.europa.eu/eusurvey/home/welcome. Survey responses of rare endocrine disease patients (or their parents and or caregivers) were gathered between late 23 December 2019 and 1 April 2020.

Before starting the survey, patients gave their consent for scientific analysis and publication of their answers. Baseline questions were related to participants’ disease area, gender, age group and membership of any patient organization. Thereafter, patients were asked to rate the importance of nine prespecified topics in scientific research on a three-point numerical priority rating scale with 1 corresponding to “very important”, 2 to “important” and 3 to “less important”. The prespecified topics were heritability, fertility, tiredness, ability to participate in sports, ability to work, ability to take part in social activities, daily medicine intake, sleep quality and physical discomfort. In addition, patients were asked what disease-related subjects “keep them up at night” and were given the possibility to provide additional suggestions for important research topics in open fields. Finally, they were asked to indicate the most important sources of scientific information on their disease via check boxes.

After distribution of the survey by the Swedish Thyroid patient advocacy group, a disproportionately high number of responses containing references to hypothyroidism were received. This raised suspicion that not only patients with a rare thyroid disease responded, but also patients who have more common thyroid disorders. Therefore, responses by Swedish thyroid patients not specifically mentioning rare thyroid disorders were excluded from analysis.

### Statistical analysis of quantitative data

Europe was divided into four regions in concordance with the definition used by the United Nations Statistics Division [5]. Statistical analysis of quantitative data was performed using IBM SPSS statistics 25.0 (IBM Corp. Released 2017. Armonk, NY, USA). Descriptive statistics were used to describe and present the cohort. To assess differences in rated importance between research topics, paired samples *t*-tests were performed. To assess the difference of rated importance of topics between groups (e.g., sex, MTG, region), one-way ANOVA tests were performed with post hoc *t*-tests with Bonferroni correction; significance was taken at *p* < 0.05.

### Analysis of open field data

Google Translate Application Programming Interface with automatic source language detection was used to translate all answers to English. During analysis, all responses to an open field question were combined and weighted equally. Thereafter, two survey questions concerning urgent research areas were combined. To determine the most important topics overall, we used a topic modelling technique called non-Negative Matrix Factorization [6]. This algorithm computes optimal clustering of words used in survey responses, representing underlying topics. Words are weighted with “Term Frequency—Inverse Document Frequency” score: a statistical measure relevance. Number of word appearances (i.e., its term frequency) is multiplied by how common the word is in all responses (i.e., its inverse document frequency). Thereby, words that are common to every survey response (like “the” or “and” but also words relating to survey questions) get a low weight and do not affect the topic modelling. To ensure that frequently occurring words do not impact topic modelling, highly frequent words in English (also coined stop words) are excluded. Non-Negative Matrix Factorization does not determine the number of topics automatically, but clusters words optimally given the number of topics “k”. “k” was determined by choosing the number of topics for which average coherence within topics was highest. Topic coherence was measured using TC-W2V metric [7]. This metric requires words to be mapped to numeric vectors based on the context of the word [8]: words with similar meanings end up with similar numerical representations and how close these numerical vectors are (i.e., their cosine similarity) can be used to measure how similar the words are. The average cosine similarity of words belonging to a topic is its TC-W2V coherence. Topic labels were assigned manually by exploring words with the highest weights for that topic.

Due to small size of the data, topic modelling could not be used per MTG. Instead, we used the Kullback–Leibler divergence for Informativeness and Phraseness metric [9] to identify the most distinctive phrases (maximum four words) per MTG. Kullback–Leibler divergence is a measure from information theory that estimates the amount of information that is lost if probability distribution A is used to approximate probability distribution B. To this end, the survey responses (distribution B) are compared to a background corpus (distribution A): a large set of Wikipedia pages (circa 3.5 million words) [10] provided by Verberne et al. [9]. The informativeness of a term is then those terms for which the expected loss of information is highest, i.e., terms that are (much) more frequent in responses than in the background collection. In order to extract multi-word phrases instead of single words, the phraseness of combinations of words is calculated. Phraseness is a score for how likely terms are to occur together compared to how likely they are to occur alone. The KLIP metric balances phraseness and informativeness with the parameter γ, which was set at 0.8, as recommended by Tomokiyo et al. To allow for grouping of different inflected forms of words, we used lemmatisation, i.e., the linguistic process of reducing words to their dictionary form or lemma (e.g., sleeping to sleep or apples to apple). To improve the detection of phrases, punctuation was excluded, and terms were not allowed to start or end with stop words. Additionally, terms were not allowed to span across different responses and had to occur at least two times to be included. Finally, three- and four-word combinations that occurred more than once in the answers of the same person were removed in order to avoid biasing the results towards one person’s opinion. Tables with outcomes of the analysis are presented in the Supplementary files. The terms were checked by an endocrinologist for relevance and direct references to diseases or suggested topics were omitted from the results section.

## Results

### Demographics

**Fig. 1:**
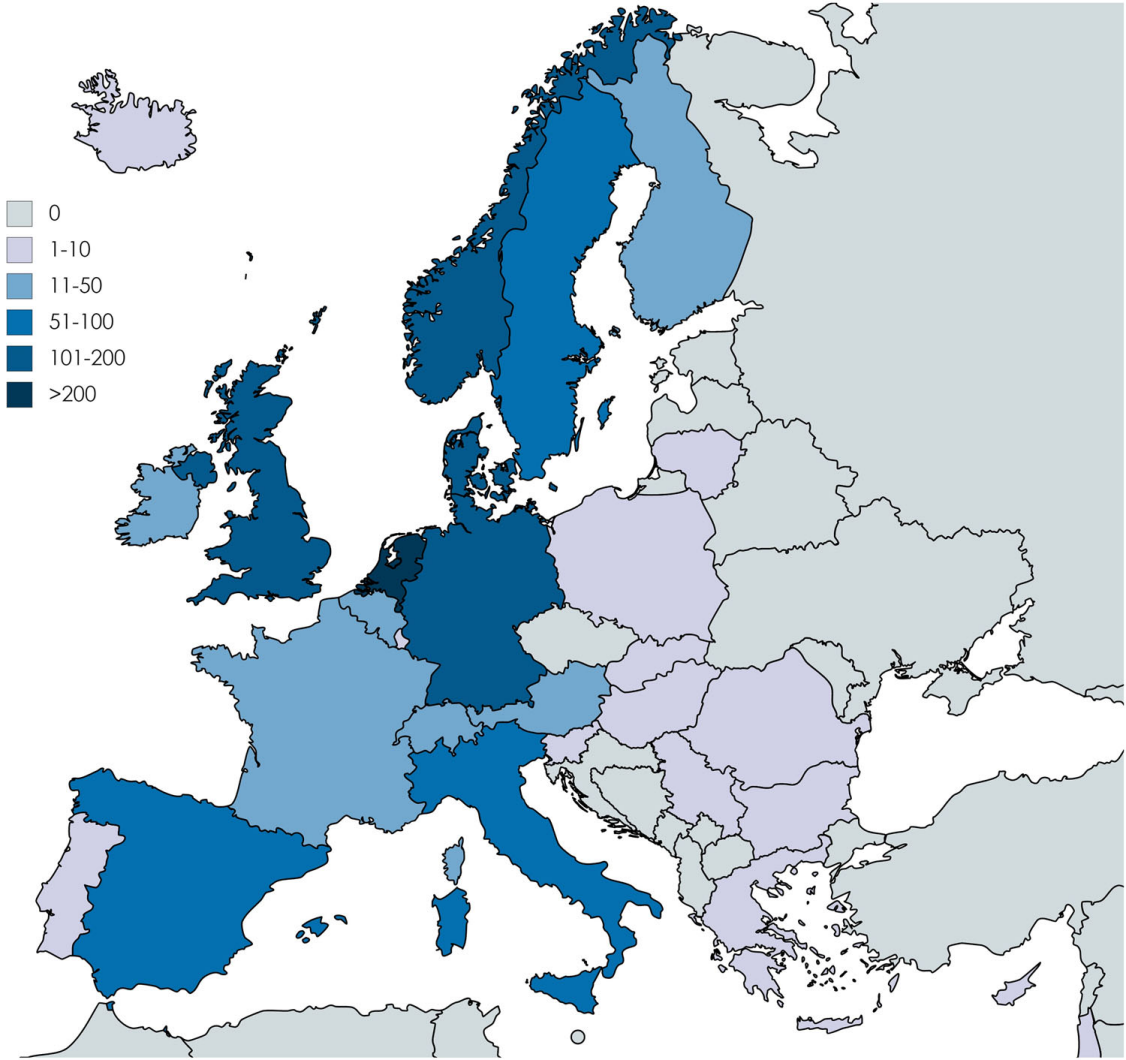
Absolute number of responses per country

After data cleaning, 1378 survey responses were analyzed. Demographic data are presented in Table 1 and Fig. 1. Most respondents were female (*n* = 1084, 79%); the majority of respondents were members of a patient advocacy group (69%). Southern (11%) and especially Eastern Europe (2%) were poorly represented. Most responses were received from patients of the Adrenal (39%), Pituitary (29%) and Thyroid (13%) MTGs, whereas the Genetic disorders of glucose and insulin homoeostasis MTG had the lowest representation (2%). In spite of additional effort, no extra subjects could be identified for underrepresented MTGs.

**Table 1:** Demographic data of survey responders

	*n* (%)
Gender	
Female	1084 (78.7)
Male	284 (20.6)
Prefer not to say	10 (0.7)
Age	
<10	20 (1.5)
10–18	22 (1.6)
19–30	110 (8.0)
31–40	231 (16.8)
41–50	388 (28.2)
51–60	346 (25.1)
61–70	198 (14.4)
>70	63 (4.6)
MTG	
Adrenal	539 (39.1)
Disorders of calcium and phosphate homoeostasis	46 (3.3)
Genetic disorders of glucose and insulin homoeostasis	22 (1.6)
Genetic endocrine tumour syndromes	75 (5.4)
Growth and genetic obesity syndromes	48 (3.5)
Pituitary	398 (28.9)
Sex development and maturation	70 (5.1)
Thyroid	180 (13.1)
Region	
Eastern Europe	26 (1.9)
Northern Europe	649 (47.1)
Southern Europe	146 (10.6)
Western Europe	536 (38.5)
Non-Europe	21 (1.5)
Respondent is	
Patient	1219 (88.5)
Caregiver	159 (11.5)
Member of patient advocacy group	944 (68.5)

*MTG* main thematic group

### Priorities of suggested research topics

**Fig. 2:**
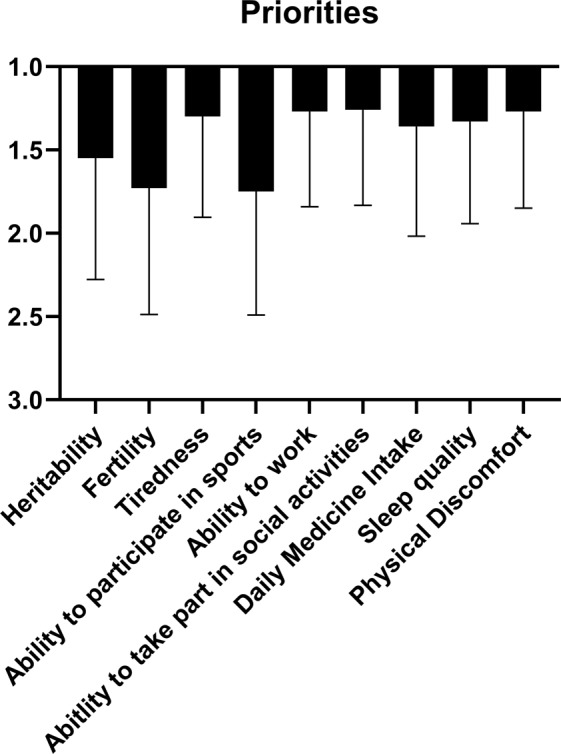
Mean priorities with standard deviations as rated by respondents from 1 to 3 in order of prioritization, 1 being the most important priority

Patients rated ability to take part in social activities as the most important suggested research topic (priority score: 1.26 ± 0.57), closely followed by ability to work (1.27 ± 0.57) and physical discomfort (1.27 ± 0.58). A second tier of topics consisted of tiredness (1.30 ± 0.61), sleep quality (1.33 ± 0.61) and daily medicine intake (1.36 ± 0.66). Patients had the least interest in research concerning heritability (1.55 ± 0.73), fertility (1.73 ± 0.76) and ability to participate in sports (1.75 ± 0.74) (Fig. 2).

Female respondents were significantly more interested in research on fertility than male respondents (priority score: 1.70 ± 0.76 vs. 1.86 ± 0.75, *p* = 0.008). There were no further gender-specific differences in the interest on the suggested research topics (Supplementary Table S1). Respondents from the Adrenal, Pituitary and Thyroid MTGs were more interested in research on tiredness than others (*p* < 0.001). Patients from the Adrenal MTG were also more interested in research on daily medicine intake than other respondents and rated this as the most important suggested research topic (1.21 ± 0.54, *p* < 0.001). Respondents of Sex Development and Maturation MTG were more interested in research on fertility (1.30 ± 0.58, second most important topic, *p* < 0.001) and less interested in research on heritability (1.97 ± 0.85, least important topic, *p* < 0.001) than patients from other MTGs (Table 2). Respondents from Southern Europe were more interested in the ability to participate in sports than respondents from Northern Europe (*p* = 0.002). Patients from Western Europe were more interested in research on physical discomfort than patients from Northern Europe (*p* = 0.012). Comparisons between regions were, however, not corrected for the difference in representation of MTGs.

**Table 2:** Priority scores of suggested topics per MTG

	All	MTG1	MTG2	MTG3	MTG4	MTG5	MTG6	MTG7	MTG8
Heritability	1.55 ± 0.73	1.47 ± 0.67^a^	1.59 ± 0.73	1.36 ± 0.73^b^	1.44 ± 0.67^c^	1.42 ± 0.69^d^	1.62 ± 0.76^a^	1.97 ± 0.85^a,b,c,d,e^	1.55 ± 0.75^e^
Fertility	1.73 ± 0.76	1.74 ± 0.76^a^	1.93 ± 0.81^b^	1.65 ± 0.75	1.84 ± 0.78^c^	1.56 ± 0.73	1.76 ± 0.74^d^	1.30 ± 0.58 ^a,b,c,d,e^	1.78 ± 0.80^e^
Tiredness	1.30 ± 0.61	1.28 ± 0.58^a^	1.44 ± 0.69	1.57 ± 0.75	1.39 ± 0.68	1.56 ± 0.66^b^	1.23 ± 0.54^b,c^	1.54 ± 0.77 ^a,c,d^	1.25 ± 0.60^d^
Sports	1.75 ± 0.74	1.69 ± 0.75^a^	1.70 ± 0.63	1.55 ± 0.74	2.00 ± 0.76^a^	1.76 ± 0.67	1.78 ± 0.72	1.90 ± 0.78	1.76 ± 0.76
Work	1.27 ± 0.57	1.29 ± 0.59	1.17 ± 0.49	1.23 ± 0.61	1.29 ± 0.61	1.38 ± 0.61	1.23 ± 0.53	1.32 ± 0.66	1.26 ± 0.57
Social life	1.26 ± 0.57	1.28 ± 0.58	1.20 ± 0.58	1.27 ± 0.55	1.19 ± 0.49	1.28 ± 0.50	1.25 ± 0.56	1.26 ± 0.61	1.31 ± 0.63
Daily medicine intake	1.36 ± 0.66	1.21 ± 0.54^a,b,c,d^	1.33 ± 0.60	1.27 ± 0.63	1.45 ± 0.69	1.58 ± 0.75^a^	1.44 ± 0.70^b^	1.54 ± 0.74^c^	1.49 ± 0.73^d^
Sleep quality	1.33 ± 0.61	1.31 ± 0.59^a^	1.43 ± 0.70	1.38 ± 0.59	1.27 ± 0.53^c^	1.41 ± 0.69	1.31 ± 0.60^b^	1.58 ± 0.72^a,b,c^	1.31 ± 0.62^c^
Physical discomfort	1.27 ± 0.58	1.29 ± 0.60	1.13 ± 0.40	1.23 ± 0.61	1.21 ± 0.54	1.15 ± 0.36	1.27 ± 0.58	1.47 ± 0.70	1.24 ± 0.57

The superscript letters denote that mean priority scores of the marked MTGs differ significantly. MTG1: Adrenal; MTG2: Disorders of calcium and phosphate homoeostasis; MTG3: Genetic disorders of glucose and insulin homoeostasis; MTG4: Genetic endocrine tumour syndromes; MTG5: Growth and genetic obesity syndromes; MTG6: Pituitary; MTG7: Sex development and maturation; MTG8: Thyroid

*MTG* main thematic group

### What keeps patients up at night

When analyzing all responses, the most important subject was pain, mainly in joints and muscles. Other frequently mentioned subjects were chronic fatigue, quality of life, daily medication and its dosage and long-term side effects of drugs (Supplementary Table S2A).

In general, mentioned subjects were very disease specific. Adrenal MTG patients mentioned adrenal crisis as the most important subject that kept them awake. Genetic Endocrine Tumour Syndromes MTG patients mentioned uncertainty about the future and their family, while Sex Development and Maturation MTG patients are particularly worried by hearing loss. Pituitary patients frequently mentioned restless legs, adrenal crisis and blood pressure as particular reasons for concerns, whereas thyroid patients mentioned rest complaints and energy levels (Supplementary Table S3A).

### Urgent and so far insufficiently covered research topics according to patients

Patients most often mentioned better treatment, in particular medication, as a research topic they want scientists to focus on. Additionally, they request research on long-term side effects of drugs. They also want researchers to investigate quality of life, chronic fatigue and causes of rare endocrine diseases (Supplementary Table S2B).

Adrenal patients rate increased research efforts on emergency (glucocorticoid) injections and circadian rhythm urgently necessary. Patients with Genetic disorders of Glucose and Insulin Homoeostasis request research on muscle weakness and dental effects of their disease, while Genetic Endocrine Tumour Syndromes patients list gene therapy and mental health as important research topics. Mental health is also a theme suggested by pituitary patients, as well as different endocrine consequences of their disease, i.e., growth hormone (deficiency), adrenal insufficiency and diabetes insipidus, whereas patients from the Sex Development and Maturation MTG want more research on hearing loss in their disease. Thyroid patients would like more research on radioactive iodine treatment (not further specified), residual complaints (following treatment) and slow release T3. No additional research topics were identified by Growth and Genetic Obesity Syndrome patients (Supplementary Table S3B).

### Sources of information

**Fig. 3:**
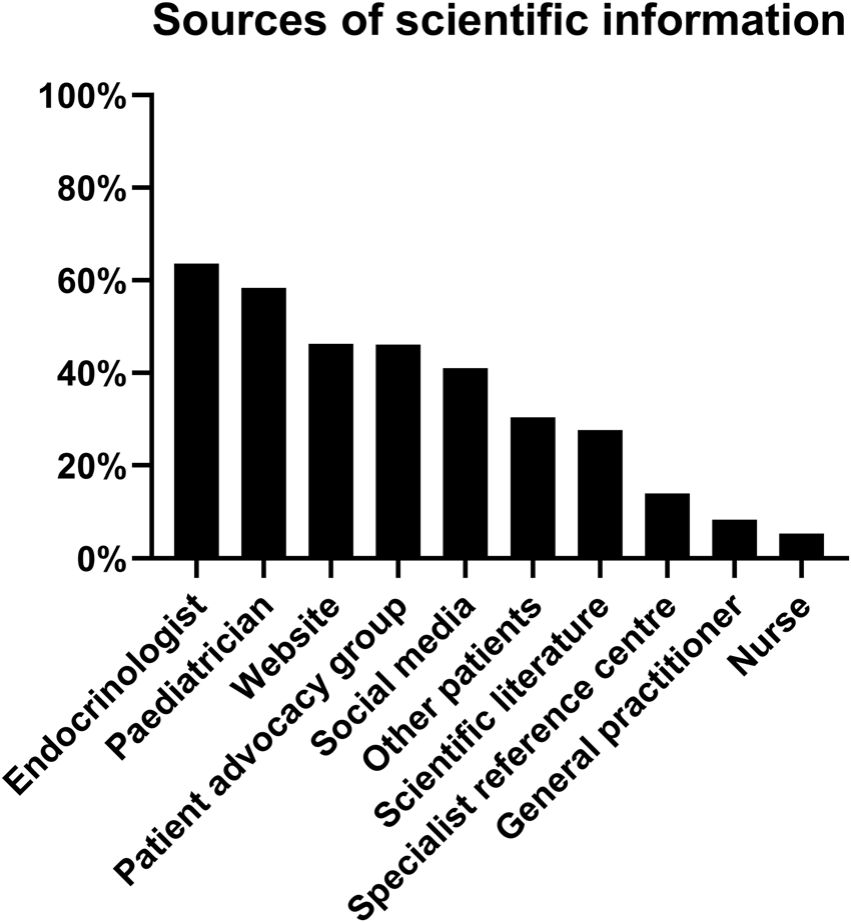
Percentage of all respondents that listed source as a used source for scientific information on their disease. For Paediatrician, only patients younger than 18 years were analyzed. Paediatrician included paediatric endocrinologist

Patients listed their treating endocrinologist as their main source of scientific information (63.6%), followed by websites (46.3%), patient advocacy groups (46.1%), social media (41.0%) and other patients (30.4%) (Fig. 3). A substantial number (27.6%) of patients listed scientific literature as a source. Less often used sources of scientific information were specialist reference centres (14.0%), general practitioners (8.4%) and (specialized) nurses (5.4%). The majority (54.8%) of respondents younger than 18 years listed their paediatrician/paediatric endocrinologist as their main source of scientific information (Fig. 3).

## Discussion

This study is the first large survey enabling rare endocrine disease patients in the world to express their opinion on unmet needs in medical research. It identifies which areas of research they prioritize and what research they deem urgently needed. From our list of nine prespecified topics of research, functional outcomes “participation in social life” and “ability to work” are considered as most important research topics by patients, irrespective of their particular condition. While some recent clinical trials have made an effort to include at least one measure on quality of life (e.g., SF-36 or AGHDA score in adult GH deficiency studies), most studies in the endocrine field focus on clinician-reported outcomes [11–15]. Identification of crucial functional outcomes from patient’s perspective is therefore a valuable observation. Additionally, patients urgently request researchers to focus on long-term side effects of medical treatment and patient-reported outcome measures, such as quality of life and mental health. These results concur with the results of pioneering studies performed in the area of variations in sexual development where a workshop was held on involvement of patients with atypical sexual development and their parents [16] and a survey among clinicians caring for these patients [17]. Clinicians rated quality of life as the most urgent research topic within this disease group [17], whereas patients and parents pointed out that quality of life studies should also consider gender development, sexual function and comorbidities. Moreover, patients also suggested research on the long-term effect of medication and on fertility preservation and reproductive technology [16]. Research priorities differ between disease groups and highlight particular characteristics of different diseases. This study also assessed patients’ main sources of scientific information. For most respondents (paediatric), endocrinologists are still the most important and accessible source of information, but the fact that over a third of patients do not list them as an informational source is of concern. In contrast, social media is an emerging source of accessible information.

A clear bias towards the best represented groups was observed, i.e., adrenal, pituitary and thyroid patients. Therefore, future research efforts should particularly target rare endocrine diseases that are underrepresented in this study. Results were also analyzed for MTGs separately, although power of quantitative comparisons and usefulness of open field analyses is more limited, given the lower number of respondents in each MTG. In open field analyses of individual MTGs, suggested outcomes had to be interpreted by endocrinologists to select useful terms and topics, which introduce interpretation bias.

During the distribution phase of this survey, we observed large differences in the landscape of patient representation in Europe. A well-established national infrastructure of patient organizations is paramount in the dissemination process of such a survey, as it allows to contact their members directly. Eurordis provided valuable support and helped us to identify a high number of patient organizations within Europe that are active on a national or regional level. This greatly increased the number of respondents. Some patient organizations were far better represented than others (e.g., Danish adrenal, German pituitary and Dutch thyroid cancer patient associations) and this presumably reflects their level of organization. For countries with no or few identified patient organizations, it was more difficult or even impossible to reach patients, resulting in clear underrepresentation, which hampered data analysis.

In the designing phase of this survey, the importance of addressing patients in their native language to increase response rates became very apparent. Therefore, considerable effort was put in translation of the survey into 22 European languages. However, we noticed that the use and literal translation of certain expressions resulted in interpretation issues. For example, the question “what keeps you up at night?” was intended to be interpreted figuratively and to identify major issues of concern for patients with respect to their disease. However, many respondents interpreted this expression literally, resulting in some unintended answers. Moreover, when analyzing open field data, all answers had to be translated back to English. We used Google Translate Application Programming Interface to do this automatically. However, this programme does not perform as well for every language and especially struggles with less common languages.

In conclusion, we report on a unique pan-European rare endocrine patient survey on unmet needs in medical research. An exceptionally high number of respondents report on unmet needs, particularly in functional outcome domains. These unmet needs differ between disease groups, and, to a lesser extent, between gender and regions. Insurmountable hurdles were encountered to reach patients residing in Eastern Europe, most probably related to lack of effective patient organization infrastructure in this region. We advise European policy makers and health care providers in these regions to invest additional research in identification of the reasons for this manifest inequality and to prioritize measures that might reduce it. However, Endo-ERN could provide aid in this matter and has this inequality on its agenda. The results of this study should be embraced by medical scientists in the design of future clinical studies in the rare endocrine disease field.

## Supplementary information

Supp Table S1

Supp Table S2

Supp Table S3

## References

[1] Azzopardi-Muscat N, Brand H (2015). Will European Reference Networks herald a new era of care for patients with rare and complex diseases?. Eur. J. Public Health.

[2] F. de Vries, M. Bruin, A. Cersosimo, C. N. van Beuzekom, S. F. Ahmed, R. P. Peeters, N. R. Biermasz, O. Hiort, A. M. Pereira. An overview of clinical activities in Endo-ERN: the need for alignment of future network criteria. Eur J Endocrinol. **183**(2), 141–148 (2020) 10.1530/EJE-20-0197 32413847

[3] Endo-ERN. Overview of specific expertise (2020), https://endo-ern.eu/activities/research-activities/

[4] Endo-ERN. Research activities (2020), https://endo-ern.eu/activities/research-activities/

[5] UNSD. UNSD methodology—geographic regions (2020), https://unstats.un.org/unsd/methodology/m49/

[6] Cichocki A, Phan A-H (2009). Fast local algorithms for large scale nonnegative matrix and tensor factorizations. IEICE Trans..

[7] O’Callaghan D, Greene D, Carthy J, Cunningham P (2015). An analysis of the coherence of descriptors in topic modeling. Expert Syst. Appl..

[8] Mikolov T, Sutskever I, Chen K, Corrado GS, Dean J (2013). Distributed representations of words and phrases and their compositionality. Adv. Neural Inf. Process. Syst..

[9] T. Tomokiyo, M. Hurst, A language model approach to keyphrase extraction. in Proceedings of ACL, Sapporo, Japan. (2003), pp. 33–40

[10] Verberne S, Sappelli M, Hiemstra D, Kraaij W (2016). Evaluation and analysis of term scoring methods for term extraction. Inform. Retrieval J..

[11] Glintborg D, Vaegter HB, Christensen LL (2020). Testosterone replacement therapy of opioid-induced male hypogonadism improved body composition but not pain perception: a double-blind, randomized, and placebo-controlled trial. Eur. J. Endocrinol..

[12] Buchfelder M, Lely A-JVD, Biller BMK (2018). Long-term treatment with pegvisomant: observations from 2090 acromegaly patients in ACROSTUDY. Eur. J. Endocrinol..

[13] Samuels MH, Kolobova I, Niederhausen M, Purnell JQ, Schuff KG (2018). Effects of altering levothyroxine dose on energy expenditure and body composition in subjects treated with LT4. J. Clin. Endocrinol. Metab..

[14] Colao A, Bronstein MD, Brue T (2020). Pasireotide for acromegaly: long-term outcomes from an extension to the Phase III PAOLA study. Eur. J. Endocrinol..

[15] Griffin SJ, Rutten GEHM, Khunti K (2019). Long-term effects of intensive multifactorial therapy in individuals with screen-detected type 2 diabetes in primary care: 10-year follow-up of the ADDITION-Europe cluster-randomised trial. Lancet Diabetes Endocrinol..

[16] C. Sanders, J. Hall, C. Sanders, A. Dessens, J. Bryce, N. Callens, M. Cools, M. Kourime, A. Kyriakou, A. Springer, L. Audi, A. Balsamo, V. Iotova, V. Mladenov, M. Krawczynski, A. Nordenskjöld, M. Rozas, H. Claahsen-van der Grinten, O. Hiort, S. Riedl, S.F. Ahmed. Involving Individuals with Disorders of Sex Development and Their Parents in Exploring New Models of Shared Learning: Proceedings from a DSDnet COST Action Workshop. Sex Dev. **12**, 225–231 (2018) 10.1159/000490081 29936513

[17] Hiort O, Cools M, Springer A (2019). Addressing gaps in care of people with conditions affecting sex development and maturation. Nat. Rev. Endocrinol..

