# Supplementary material for: Patients with rare endocrine conditions have corresponding views on unmet needs in clinical research
Source: Endocrine. 2021 Feb 3;71(3):561–8. doi: 10.1007/s12020-021-02618-z (PMC8016771; doi:10.1007/s12020-021-02618-z)
Supplement: Supplementary file 1 — Supp Table S1 [file 12020_2021_2618_MOESM1_ESM.docx]

**Supplementary table S1: priority scores of suggested topics per gender**

|  | All | Female | Male | Prefer not to say |
| --- | --- | --- | --- | --- |
| Heritability | 1.55±0.73 | 1.55±0.73 | 1.52±0.71 | 1.67±1.00 |
| Fertility | 1.73±0.76 | 1.70±0.76^a^ | 1.86±0.75^a^ | 1.33±0.50 |
| Tiredness | 1.30±0.61 | 1.29±0.60 | 1.34±0.62 | 1.33±0.50 |
| Sports | 1.75±0.74 | 1.75±0.74 | 1.75±0.74 | 1.80±0.79 |
| Work | 1.27±0.57 | 1.27±0.58 | 1.24±0.55 | 1.40±0.70 |
| Social life | 1.26±0.57 | 1.26±0.58 | 1.26±0.54 | 1.50±0.85 |
| Daily medicine intake | 1.36±0.66 | 1.35±0.65 | 1.41±0.69 | 1.10±0.32 |
| Sleep quality | 1.33±0.61 | 1.33±0.62 | 1.33±0.58 | 1.20±0.42 |
| Physical discomfort | 1.27±0.58 | 1.26±0.57 | 1.30±0.60 | 1.30±0.48 |

The superscript letters denote that mean priority score of the marked sexes differ significantly.
