# Supplementary material for: Patients with rare endocrine conditions have corresponding views on unmet needs in clinical research
Source: Endocrine. 2021 Feb 3;71(3):561–8. doi: 10.1007/s12020-021-02618-z (PMC8016771; doi:10.1007/s12020-021-02618-z)
Supplement: Supplementary file 2 — Supp Table S2 [file 12020_2021_2618_MOESM2_ESM.docx]

**Supplementary table S2**

| **A** | **Manually assigned label** | **Top 10 words for this topic** | **B** | **Manually assigned label** | **Top 10 words for this topic** |
| --- | --- | --- | --- | --- | --- |
| 1 | Pain, mainly in joints and muscles | Pain, joint, muscle, discomfort, body, muscles, management, osteoarthritis, back, bone | 1 | Addison’s disease | Addison’s, morbus, get, crisis, disease, patients, reason, stress, cure, primary |
| 2 | Chronic fatigue | Fatigue, chronic, performance, extreme, constant, insomnia, brain, causing, impact, going | 2 | Better medication | medication, better, new, adjustment, side, optimal, use, set, dosage, improved |
| 3 | Quality of life | Life, quality, expectancy, lack, reduced, span, age, daily, illness, sleeping | 3 | Thyroid cancer | thyroid, cancer, gland, consequences, food, disorders, problems, complications, low, genetics |
| 4 | Daily medication | Medication, daily, setting, take, availability, lack, proper, low, taking, adjustments | 4 | Chronic fatigue | fatigue, extreme, chronic, severe, maybe, disorders, reduce, pain, Addison’s, concentration |
| 5 | Fertility | Fertility, worries, needed, infertility, issues, imbalances, follow, pregnant, replacement, birth | 5 | Pituitary tumors | pituitary, tumor, gland, tumours, tumors, causes, symptoms, adenoma, related, surgery |
| 6 | Long term side effects of medication | Effects, side, term, long, medicine, drugs, treatment, cortisone, hydrocortisone, medications | 6 | Better treatment | treatment, better, t3, patients, medical, methods, also, quicker, rai, cancer |
|  |  |  | 7 | Secondary adrenal insufficiency | adrenal, insufficiency, secondary, glands, crisis, gland, emergency, impact, health, use |
|  |  |  | 8 | Cushing disease | disease, Cushing, Addison’s, causes, diagnosis, autoimmune, development, graves, heart, early |
|  |  |  | 9 | Long term side effects of drugs | effects, side, drugs, term, long, medication, psychological, hydrocortisone, symptoms, late |
|  |  |  | 10 | Better quality of life | life, quality, improve, impact, patients, every day, patient, social, better, chronic |
|  |  |  | 11 | Heredity | heredity, tumor, confirmation, opportunity, aches, genes, statements, libido, financial, pharmacy |
|  |  |  | 12 | Autoimmune diseases | diseases, autoimmune, rare, several, endocrine, impact, accompanying, relationship, prevention, relationships |
|  |  |  | 13 | Diabetes insipidus | diabetes, insipidus, morbidities, cortisol, measure, Addison patient, common, reason, genetic, turner |
|  |  |  | 14 | Causes of disease | cause, disorder, condition, adenoma, autoimmune, acromegaly, Cushing, genesis, toxic, prolactinoma |
|  |  |  | 15 | Hormone growth and deficiency | hormone, growth, deficiency, levels, hormones, replacement, parathyroid, disruption, psychological, cortisol |
|  |  |  | 16 | Fertility | fertility, syndrome, studies, turner, CAH, women, problems, issues, pregnancy, DSD |

Results of topic modelling of all responses. A: Answers to “What keeps you up at night?”. B: Answers to “What medical research is urgently necessary?”.
