# Supplementary material for: Patients with rare endocrine conditions have corresponding views on unmet needs in clinical research
Source: Endocrine. 2021 Feb 3;71(3):561–8. doi: 10.1007/s12020-021-02618-z (PMC8016771; doi:10.1007/s12020-021-02618-z)
Supplement: Supplementary file 3 — Supp Table S3 [file 12020_2021_2618_MOESM3_ESM.docx]

**Supplementary table S3**

| **A** | **MTG1** | **MTG2** | **MTG3** | **MTG4** |
| --- | --- | --- | --- | --- |
| 1 | side effect | side effect | loss | long term |
| 2 | quality of life | future | life | family member |
| 3 | long term | transition | work | not knowing |
| 4 | autoimmune disease | treatment | fatigue | future |
| 5 | long term side effect | pain | therapy | lack |
| 6 | adrenal crisis | management |  | condition |
| 7 | social life | disease |  | not |
| 8 | able to work | daily |  | related |
| 9 | ability to work | medication |  | care |
| 10 | addison crisis | symptom |  | long |
|  |  |  |  |  |
|  | **MTG5** | **MTG6** | **MTG7** | **MTG8** |
| 1 | Side effect | Long term | Hearing loss | Long term |
| 2 | Effect aged | Quality of life | Drug | Thyroid hormone |
| 3 | Effect | Side effect | Quality | Side effect |
| 4 | Child | Restless leg | Life | Rest complaint |
| 5 | Not | Adrenal crisis | Work | Physical discomfort |
| 6 | Health | Joint pain | Therapy | Daily life |
| 7 | Issue | Blood pressure | Effect | Energy level |
| 8 | Quality | Low blood pressure | Health | Thyroid cancer |
| 9 | Life | Long term effect | Problem | Not work |
| 10 | Obesity | Social life | Condition | Pain |
|  |  |  |  |  |
| **B** | **MTG1** | **MTG2** | **MTG3** | **MTG4** |
| 1 | Addison’ss disease | Hypo para | Gene therapy | Multiple endocrine neoplasia |
| 2 | Adrenal insufficiency | rare disease | Gene | Thyroid cancer |
| 3 | Quality of life | Muscle weakness | Therapy | Multiple endocrine |
| 4 | Autoimmune disease | Parathyroid hormone | Genetic | Genetic defect |
| 5 | Side effect | Long-term effects | Research | Non functioning |
| 6 | Long term | Better | Disease | Long term |
| 7 | Adrenal gland | Treatment | Cell | Endocrine neoplasia |
| 8 | Secondary adrenal insufficiency | Effect | Syndrome | Gene therapy |
| 9 | Emergency injection | Dental | Congenital | Mental health |
| 10 | Circadian rhythm | Long-term | Glucose | Thyroid |
|  |  |  |  |  |
|  | **MTG5** | **MTG6** | **MTG7** | **MTG8** |
| 1 | Growth hormone | Side effect | Turner syndrome | Thyroid cancer |
| 2 | Turner syndrome | Growht hormone | Young age | Radioactive iodine |
| 3 | Genetic | Adrenal insufficiency | Hearing loss | Autoimmune disease |
| 4 | Growth | Quality of life | Quality of life | Side effect |
| 5 | Hormone | Long term | Klinefelter syndrome | Rest complaint |
| 6 | Not | Secondary adrenal insufficiency | Growth hormone | Quality of life |
| 7 | Obesity | Pituitary tumor | Syndrome | Slow release T3 |
| 8 | Behaviour | Mental health | Disorder | Residual symptom |
| 9 | Social | Diabetes insipidus | Turner | Slow release |
| 10 | Therapy | Pituitary gland | Life | Treatment with radioactive iodine |

Top 10 results of phrases used in open field responses per MTG. A: Answers to “What keeps you up at night?”. B: Answers to “What medical research is urgently necessary?” MTG: Main Thematic Group. MTG1: Adrenal, MTG2: Disorders of calcium & phosphate homeostasis, MTG3: Genetic disorders of glucose & insulin homeostasis, MTG4: Genetic endocrine tumour syndromes, MTG5: Growth & genetic obesity syndromes, MTG6: Pituitary, MTG7: Sex development & maturation, MTG8: Thyroid.
